# Role of Chemoablation Using UGN-101 in Upper Tract Urothelial Carcinoma: A Systematic Review and Meta-Analysis of Available Evidence

**DOI:** 10.5152/tud.2024.23215

**Published:** 2024-03-01

**Authors:** Abdalla Ali Deb, Pragnitha Chitteti, Naufal Naushad, Wael Asaad, Steve Leung, Alice Hartley, Hosam Serag

**Affiliations:** 1 Department of Urology, James Cook University Hospital, Middlesbrough, UK; 2 Department of Urology, North Tees University Hospital, Stockton, UK; 3 Department of Urology, Western General Hospital, Edinburgh, UK; 4 Department of Urology, South Tyneside and Sunderland NHS Foundation Trust, Sunderland, UK; 5 Department of Urology, University Hospitals Birmingham, Birmingham, UK

**Keywords:** Upper tract urothelial cancer, chemoablation, mitomycin, meta-analysis

## Abstract

**Objective:**

To examine the safety and efficacy of chemoablation using UGN-101 in patients with upper tract urothelial cancer (UTUC).

**Methods:**

We conducted a systematic search through 7 databases/registries to identify key observational and experimental studies reporting either the efficacy or safety of UGN-101 in UTUC patients regardless of the risk or grade of the disease. The outcomes included efficacy (complete/partial/no response, survival, death, recurrence, or progression) and safety endpoints. All meta-analyses were conducted through STATA. The prevalence rate and its 95% CI were pooled across studies. A subgroup meta-analysis was conducted on follow-up. The quality was assessed using the Newcastle Ottawa Scale. Twenty studies (1051 patients) were analyzed.

**Results:**

Complete response was reported in 49% (39%-60%) of cases, and 5% (0%-15%) had disease progression. Treatment cessation was reported in 13% (3%-27%) of patients. Four percent of cases needed radical nephroureterectomy. Recurrence and death occurred in 14% (7%-23%) and 6% (2%-10%) of patients. Complications occurred in 63% (39%-85%), the majority of which were of grades I, II, and III. Ureteral stenosis was the most common complication accounting for 35% of cases. Chemoablation-related complications occurred more than procedure-related ones. Based on available evidence, the intracavitary instillation of UGN-101 gel provides an alternative therapeutic option for upper tract urothelial cancer.

**Conclusion:**

Chemoablation provides good clinical outcomes in terms of complete response, disease progression and recurrence, and the need to undergo nephroureterectomy. Complications were encountered in more than half the population; however, most of them were of low grades.

**Main Points**

- Chemoablation with UGN-101 is associated with good pathological response and lower potentiality for disease progression.
- The need for radical nephroureterectomy post-mitomycin C administration is rare.
- More than 1 in every 2 patients experience complications.

## Introduction

Upper tract urothelial cancer (UTUC), a distinct subset of urothelial cancer, is characterized by unique epidemiological, pathological, and clinical features that differentiate it from its more common counterpart, bladder cancer. Although uncommon compared to bladder cancer, it has an incidence rate that ranges 0.7%-1.7% after a prior diagnosis with bladder cancer.^1,2^ In a recent systematic review, its incidence has been reported to range from 0.75% to as high as 6.4% following cystectomy.^3,4^ The management of UTUC is predominantly surgical, either via kidney-sparing methods such as laser ablation for low-risk cancer, or radical nephroureterectomy for high-risk cases.^5,6^ Surgical intervention, even if endoscopic, is not without risk, especially in this patient cohort which is frequently co-morbid. Due to the relatively low number of patients, there are limited studies in this field, and as a result, there are no clear guidelines to guide treatment decisions and to identify which treatment option provides better outcomes in terms of safety and efficacy.^7^ Available therapeutic options, through the intracavitary approach, are limited for UTUC patients. These options include BCG or MMC as the most common ones.^8^ However, the use of BCG has been associated with higher-grade disease, further warranting the need for nephroureterectomy in these patients.^9^

The use of MMC for the topical instillation following resection of superficial bladder cancer and nephroureterectomy has shown efficacy in reducing the recurrence of low-grade diseases,^10^ albeit with limited evidence for its effectiveness in the ureter or renal pelvis. Recent studies have explored the potential of intracavitary therapy with MMC for low-grade UTUC, proposing that it may decrease disease recurrence and/or progression through various administration approaches,^11^ including retrograde, antegrade, or reflux methods, although the latter is considered suboptimal since the drug does not often reach the renal pelvis.^7^ Despite limited evidence supporting the primary use of topical MMC in the upper tracts,^12^ the development and testing of an MMC gel formulation, UGN-101,^13,14^ have led to promising clinical outcomes, earning it Fast Track and Orphan Designation status from the FDA.^15^ The OLYMPUS trial, a phase III clinical trial of UGN-101, reported a 59% complete response rate 4 weeks post treatment, indicating MMC’s potential as a kidney-sparing alternative for UTUC patients with low-grade disease,^16^ although disease recurrence was observed in 8 out of 41 patients at 12 months.^17^

Given the paucity of data, we are conducting this early-stage systematic review and meta-analysis to study the efficacy and safety of chemoablation in UTUC with an emphasis on its role in different demographic and tumor characteristics. The findings driven from this meta-analysis will be hypothesis-generating rather than providing conclusive evidence.

## Material and Methods

### Study Design and Protocol Registration

This research was conducted per the guidelines provided for systematic reviews and meta-analysis in the preferred reporting items for systematic reviews and meta-analyses (PRISMA) checklist. The study protocol was previously registered on PROSPERO database on April 3, 2023. The protocol registration number is CRD42023410456.

The design of this review followed the PICOS framework.^18^ The population included patients with UTUC only; the intervention included chemoablation (UGN-101 neoadjuvant instillation); a comparison group was not included; the outcomes included both efficacy and safety endpoints; the study design included both experimental (i.e., single-armed clinical trials) and observational (i.e., prospective and retrospective cohort, case–control, and cross-sectional) studies.

### Database Search

On February 10, 2023, a systematic search was conducted across 6 main medical databases: PubMed, Scopus, Web of Science, EBSCOhost – Academic Search Complete, Cochrane Central Register of Controlled Trials (CENTRAL), and clinicatrials.gov. We also searched the first 200 studies from Google Scholar to include the Grey Literature as well. The selected number was chosen following the recent guidelines.^19^ A manual search process was also conducted to ensure the inclusion of any potentially missing articles. This step was done at 3 levels: the first included the reading of the reference list of each of the finally included articles, the second included searching for “similar articles” on PubMed, and the final one included conducting a random search on Google platform using our keywords.

The following keywords and search terms were used to retrieve relevant articles to our PICOS framework: (Chemoablation OR UGN-101 OR “mitomycin gel” OR “mitomycin-containing”) AND (“upper tract” OR “upper urinary tract”) AND (“urothelial cancer” OR “urothelial carcinoma*” OR “urothelial neoplasm” OR “transitional cell carcinoma”). A search query was developed and modified as per each database (Supplementary Table 1).

### Eligibility Criteria and Outcome Measures

The inclusion criteria include the following: (1) original articles, (2) including patients with UTUC who received chemoablation or UGN-101 MMC-containing gel, and (3) reporting either efficacy or safety endpoints. Noteworthy, studies were selected irrespective of the language of the original publication. The efficacy endpoints included: clinical response to chemoablation in the form of complete response (CR), partial response (PR), or no response (NR) and other clinical outcomes such as survival, mortality/death, and disease recurrence or progression to a higher grade. The safety endpoints included any complications or adverse events associated with either MMC instillation or the overall procedure.

The exclusion criteria included any of the following: (1) case reports and case series (<5 cases), (2) non-original research (i.e., reviews, commentaries, guidelines, editorials, correspondence, letters to editors, etc.), (3) duplicated records or records with overlapping datasets, and (4) studies with irrelevant ‘or unextractable’ data.

### Study Selection

After the database search, all of the retrieved citations were imported into EndNote for reference organization, identification, and duplicate removal. Citations were exported to an Excel sheet for study selection. The screening sheet included the following points: authors’ names, year of publication, the DOI, the title, and the abstract of each study. Two authors carried out the screening process in 2 stages: title/abstract and full-text screening. If both authors encountered some differences, the senior author was consulted to reach an agreement.

### Data Extraction and Risk of Bias Assessment

The senior author designed the data extraction sheet using Microsoft Excel. The sheet consisted of 3 different domains. The first one included the following items regarding both the study and included patients: the last name of the first author, the year of publication, the country where the study was conducted, the design of included study, the age and gender, and the characteristics of UTUC (i.e., history, location, and size) and MMC instillation (i.e., approach and maintenance). The second one included the main outcomes (efficacy and safety endpoints), as stated above. The third one included the quality domains that were assessed in each study using the Newcastle Ottawa Scale (NOS). Each study is therefore assessed at 3 levels: selection (4 points), comparability (2 points), and outcome (3 points). Both data extraction and quality assessment were performed by 2 authors, and the senior author was consulted whenever needed to solve any differences amongst authors.

### Data Synthesis

STATA Software version 17.0 (StataCorp.; USA) was used to conduct all statistical analyses. Since all of the meta-analyses were conducted on single-armed studies, the metaprop command was used to pool the effect size (ES) across all studies along with its 95% confidence interval (CI). Due to encountered statistical heterogeneity (*I*^2^ statistic >50% and *P* value < .05), the random-effects model was used. A subgroup meta-analysis was performed to determine the effect-modifying role of the follow-up time on each of the assessed outcomes. Publication bias could not be assessed due to the low number of studies in each of the conducted analyses (<10 studies).

## Results

## Study Selection Results

**Figure 1.**
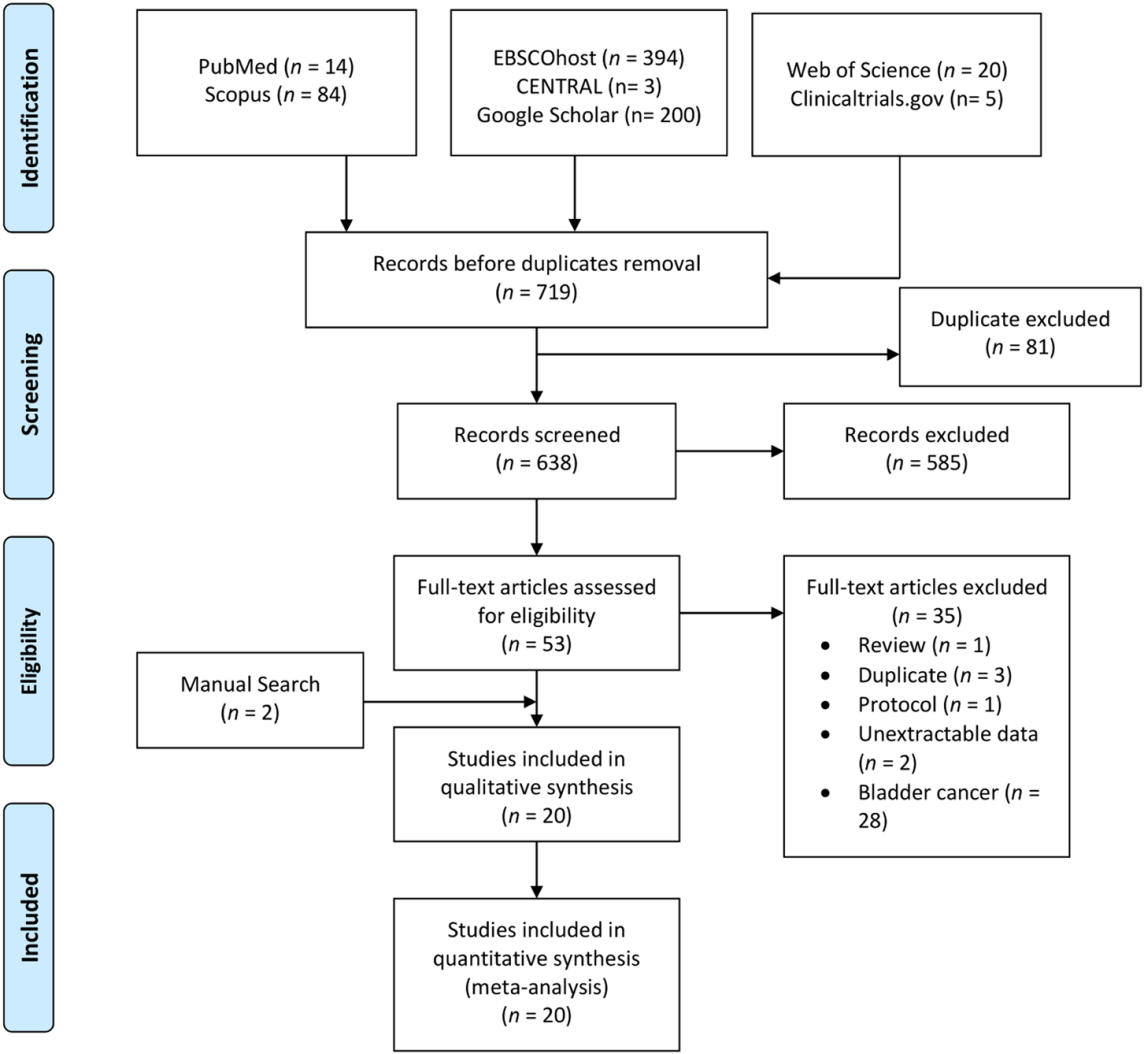
A PRISMA diagram showing the study selection process in this systematic review.

The details of the study selection process are illustrated in Figure 1. A total of 719 studies were identified through the database search, all of which were imported into EndNote for duplicate identification and removal. Afterward, 638 studies were included in the initial title and abstract screening phase. Fifty three studies were eligible for full-text screening. The full text of all eligible articles was found. Thirty-five articles were ruled out during the full-text screening phase for the following reasons: review article (n = 1), duplicated article (n = 3), study protocol (n = 1), article with unextractable data (n = 2), and articles reported bladder cancer instead of UTUC (n = 28). Finally, 2 articles were identified through the manual search, resulting in an overall number of included studies of 20.^15-17,20-36^

### Characteristics of Included Studies

The baseline characteristics of included studies and patients are summarized in Table 1. A total of 1051 UTUC patients were analyzed. The sample size among included studies ranged from as low as 8 patients to as high as 132 patients. The age and gender of included patients varied among included studies. The majority of included studies reporting the efficacy of chemoablation in UTUC were conducted in the United States (n = 15) followed by the United Kingdom (n = 3), Spain (n = 1), and Palestine (n = 1), respectively. In terms of study design, 12 of the included articles were retrospective cohort studies and the remaining 8 articles were phase III clinical trials, the majority of which (1 original trial and 4 secondary analytical studies) refer to the OLYMPUS trial but each at a different follow-up timepoint. The characteristics of UTUC and MMC instillation implemented in each study is provided in Table 2. Importantly, the definition criteria reported by included studies regarding complete and partial response are summarized in Table 1.

## Risk of Bias Assessment Results

The results of the quality assessment of each study in each of the assessed domains are provided in Table 3. Eighteen studies had fair overall quality and the 2 remaining studies had poor quality. Importantly, none of the included studies had good overall quality. The “outcome” domain was the main part in which quality was affected.

### Clinical Response to Chemoablation in UTUC

**Figure 2.**
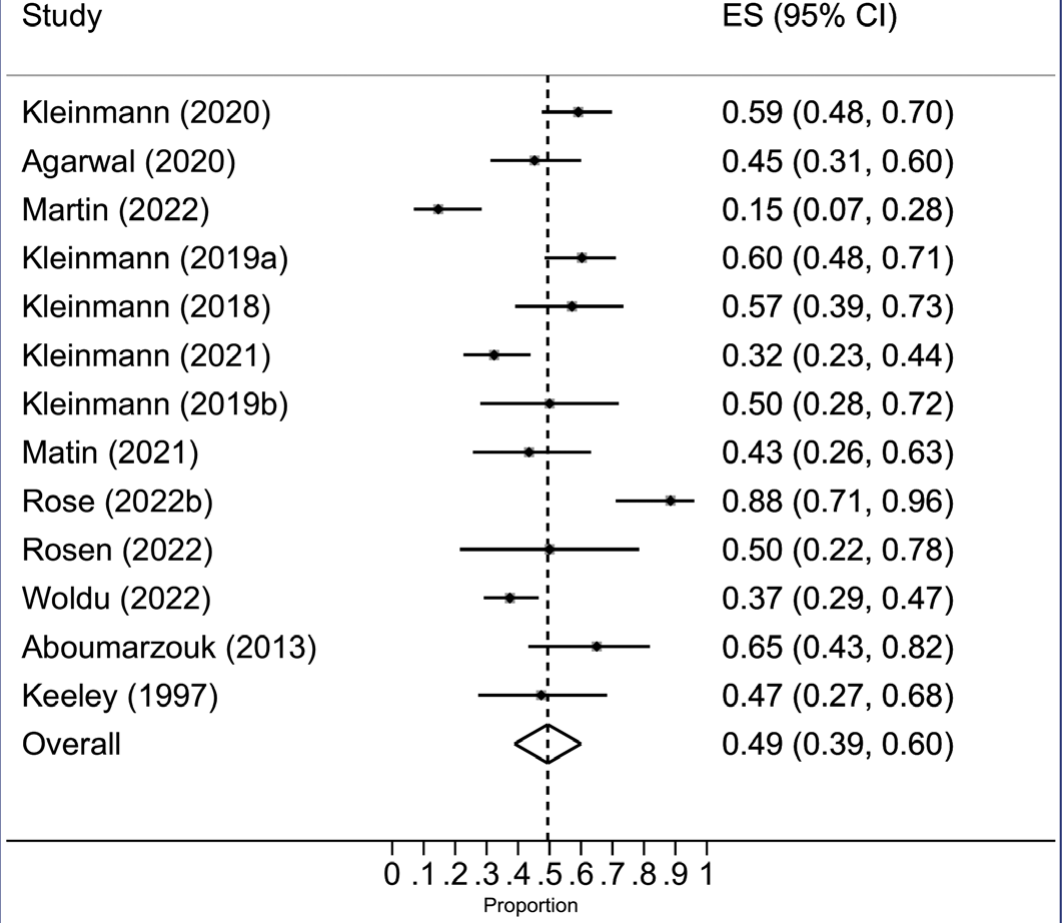
A forest plot showing the pooled prevalence of complete response following UGN-101 therapy in UTUC patients.

**Figure 3.**
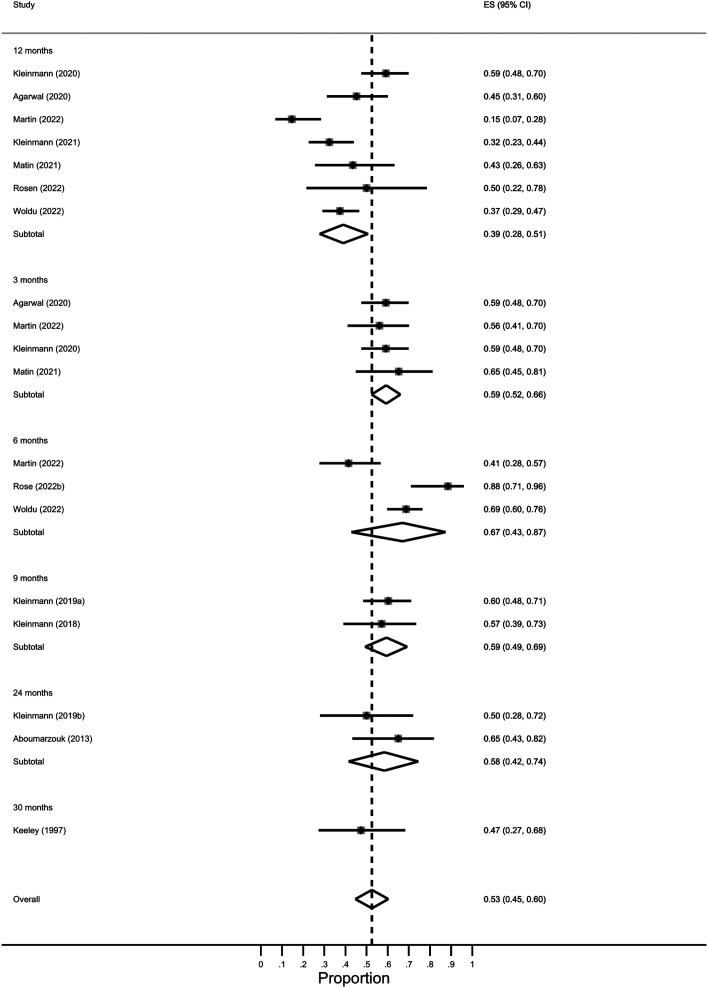
A forest plot showing the pooled prevalence of complete response following UGN-101 therapy in UTUC patients based on follow-up.

The rate of complete response (CR) of chemoablation was reported in a total of 13 studies. The meta-analysis revealed an overall prevalence rate of CR of 49% (95% CI: 39%-60%) (Figure 2). The duration of follow-up was a significant effect-modifier, changing the prevalence of CR in different time points (Figure 3). The rate of CR was highest during the earliest follow-up time points such as 3 months (ES = 59%; 95% CI: 52%-66%) and 6 months (ES = 67%; 95% CI: 43%-87%). However, at 12 months of follow-up, the rate of CR drastically declined to 39% (95% CI: 28%-51%). During long-term follow-up, the rate of CR increased again; however, these data are based on a limited sample size.

**Supplementary Figure 1.**
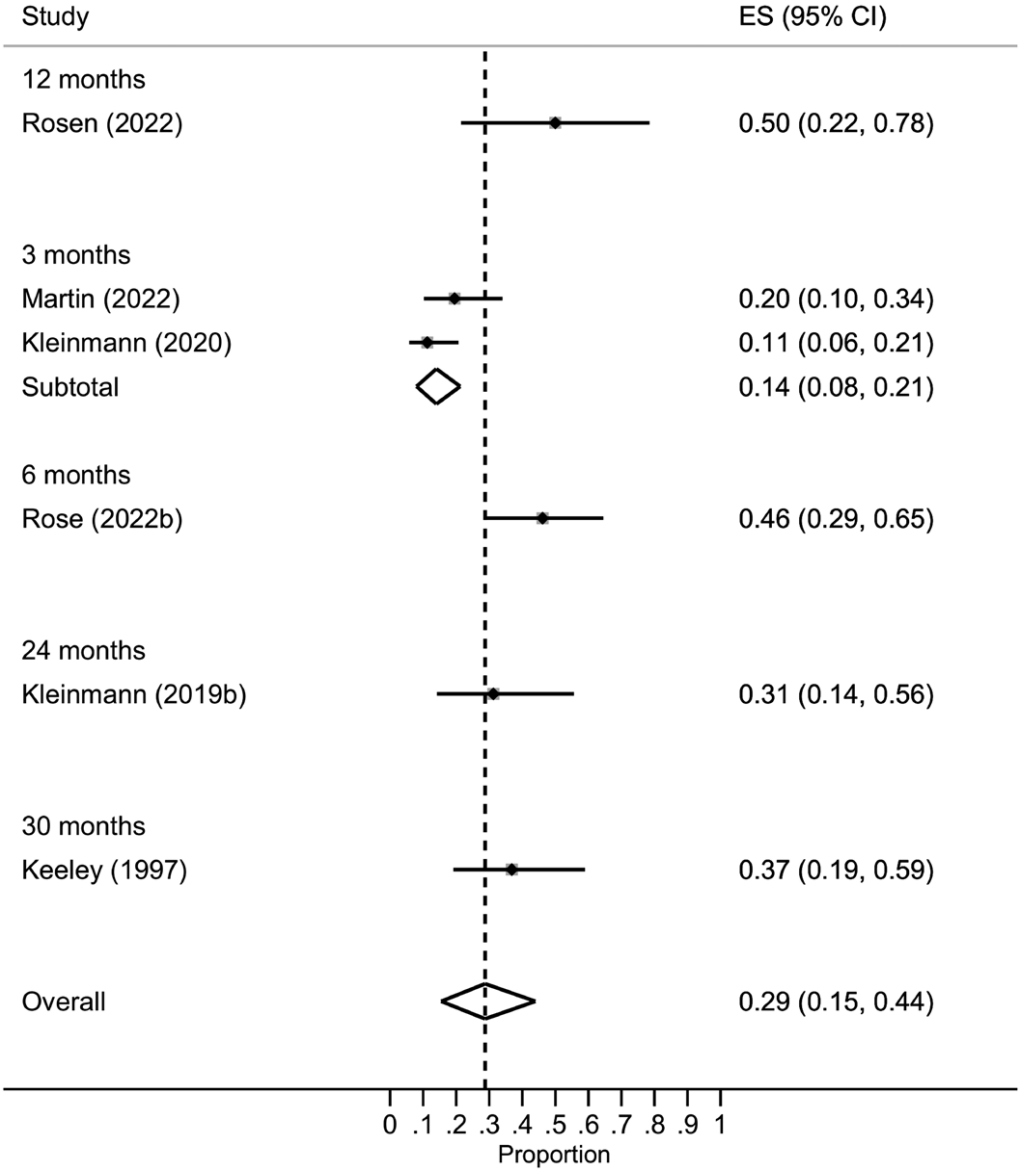
A forest plot showing the pooled prevalence of partial response following UGN-101 therapy in UTUC patients.

In terms of partial response (PR) following chemoablation, 6 studies were analyzed (Supplementary Figure 1). The meta-analysis revealed an overall prevalence rate of 29% (15%-44%). The rate of PR differed substantially in different follow-up timepoints; however, due to the limited number of studies in each timepoint, the findings that can be drawn from this analysis are limited.

**Supplementary Figure 2.**
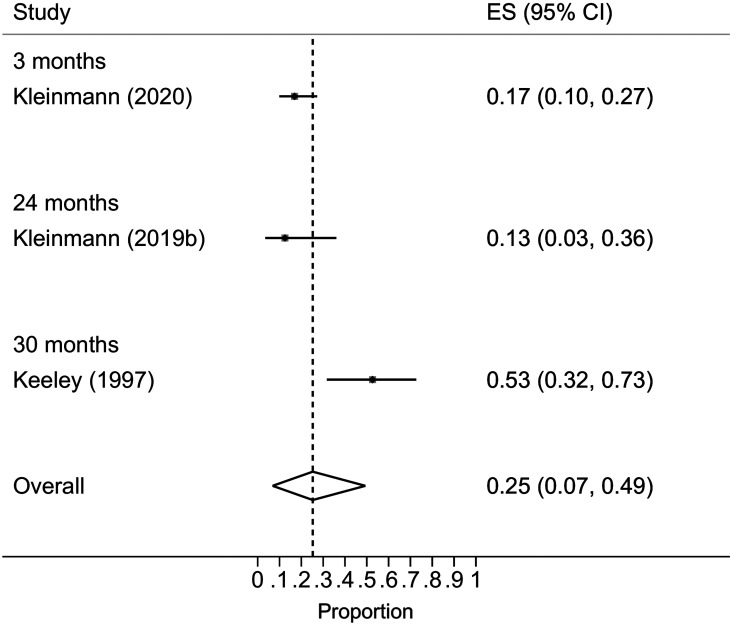
A forest plot showing the pooled prevalence of no response following UGN-101 therapy in UTUC patients.

Three studies reported the rate of no response (NR) after chemoablation (Supplementary Figure 2). The meta-analysis showed a rate of NR of 25% (95% CI: 7%-49%). Meanwhile, the data that can be drawn from the subgroup analysis based on the follow-up timepoint are limited.

### Clinical Outcomes of Chemoablation in UTUC

#### Treatment Stoppage/Cessation:

**Supplementary Figure 3.**
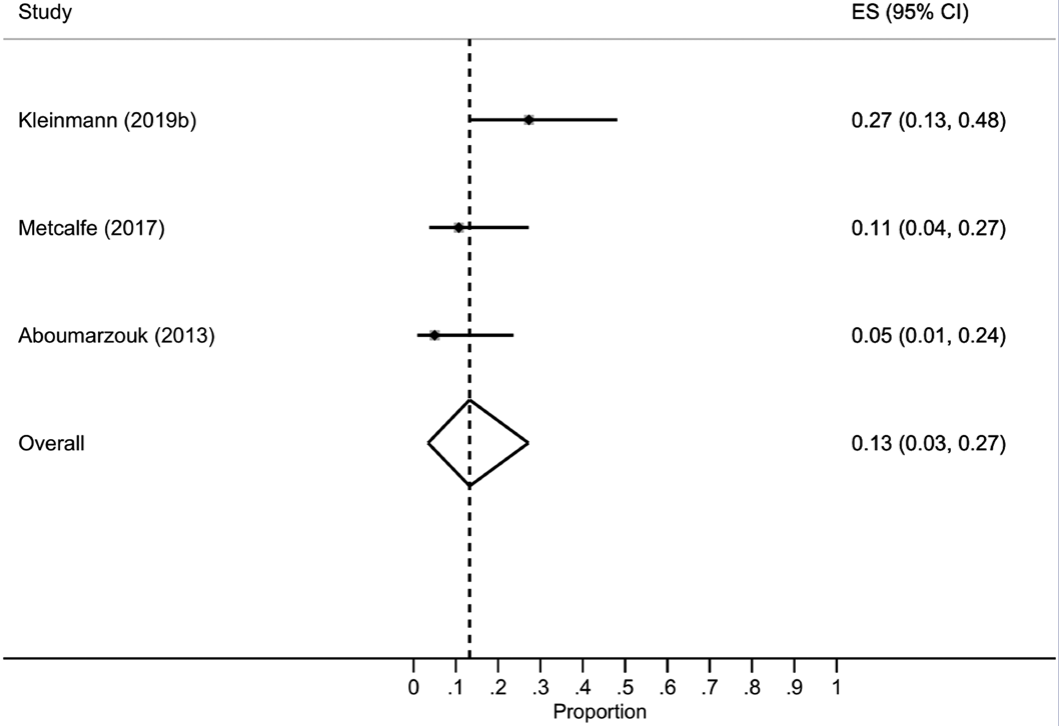
A forest plot showing the pooled prevalence of treatment cessation following UGN-101 therapy in UTUC patients.

Three studies reported the rate of chemoablation therapy cessation among UTUC patients (Supplementary Figure 3). The meta-analysis revealed an overall prevalence of treatment cessation of 13% (95% CI: 3%-27%). Due to limited data, a subgroup meta-analysis based on the duration of follow-up was not feasible.

### Progression to Invasive Disease (i.e., High or Intermediate Grade)

**Figure 4.**
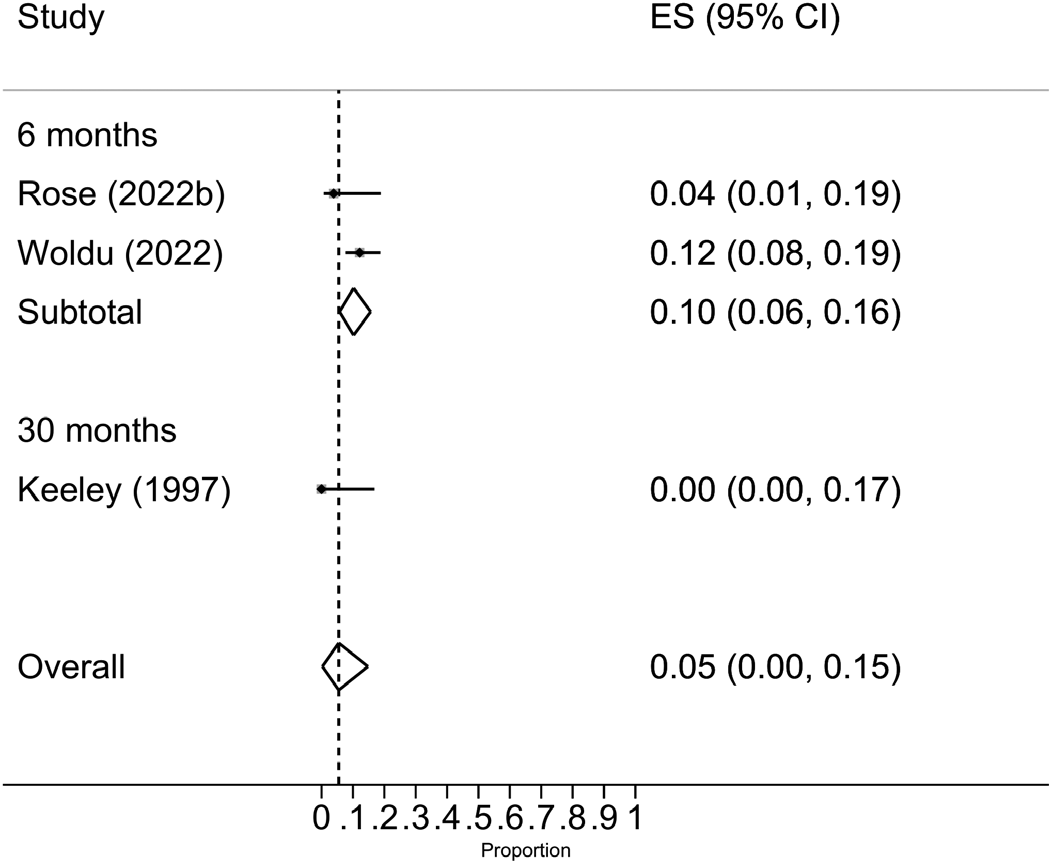
A forest plot showing the pooled prevalence of disease progression following UGN-101 therapy in UTUC patients.

Three studies reported the progression of UTUC following chemoablation therapy (Figure 4). The overall prevalence of disease progression was 5% (95% CI: 0%-15%). A noticeable difference in the rate of disease progression was noted based on the duration of follow-up time. For instance, at 6 months, the rate of disease progression was 10% (95% CI: 6%-16%) while it was 0% (95% CI: 0%-17%) at 30 months.

#### The Need for Nephroureterectomy:

**Figure 5.**
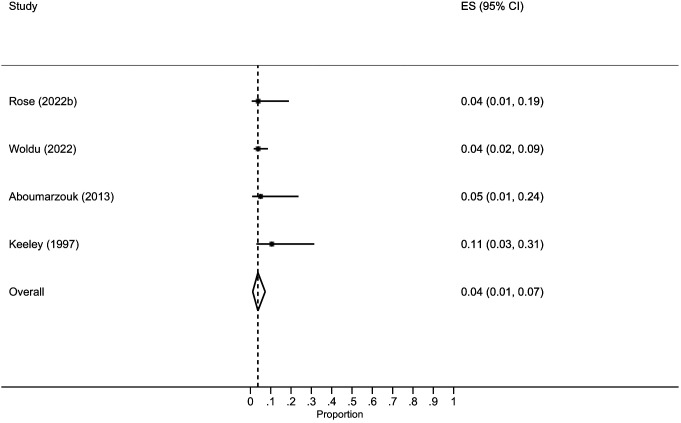
A forest plot showing the pooled prevalence of patients undergoing radical nephroureterectomy following UGN-101 therapy in UTUC patients.

Four studies reported the rate of patients who required nephroureterectomy following chemoablation therapy for UTUC (Figure 5). The meta-analysis showed an overall rate of 4% (95% CI: 1%-7%). A subgroup meta-analysis based on the follow-up duration was not feasible due to the lack of enough number of studies in each subgroup. All of these patients underwent nephroureterectomy due to disease progression (high-grade disease).^36^

### Death/Mortality

**Figure 6.**
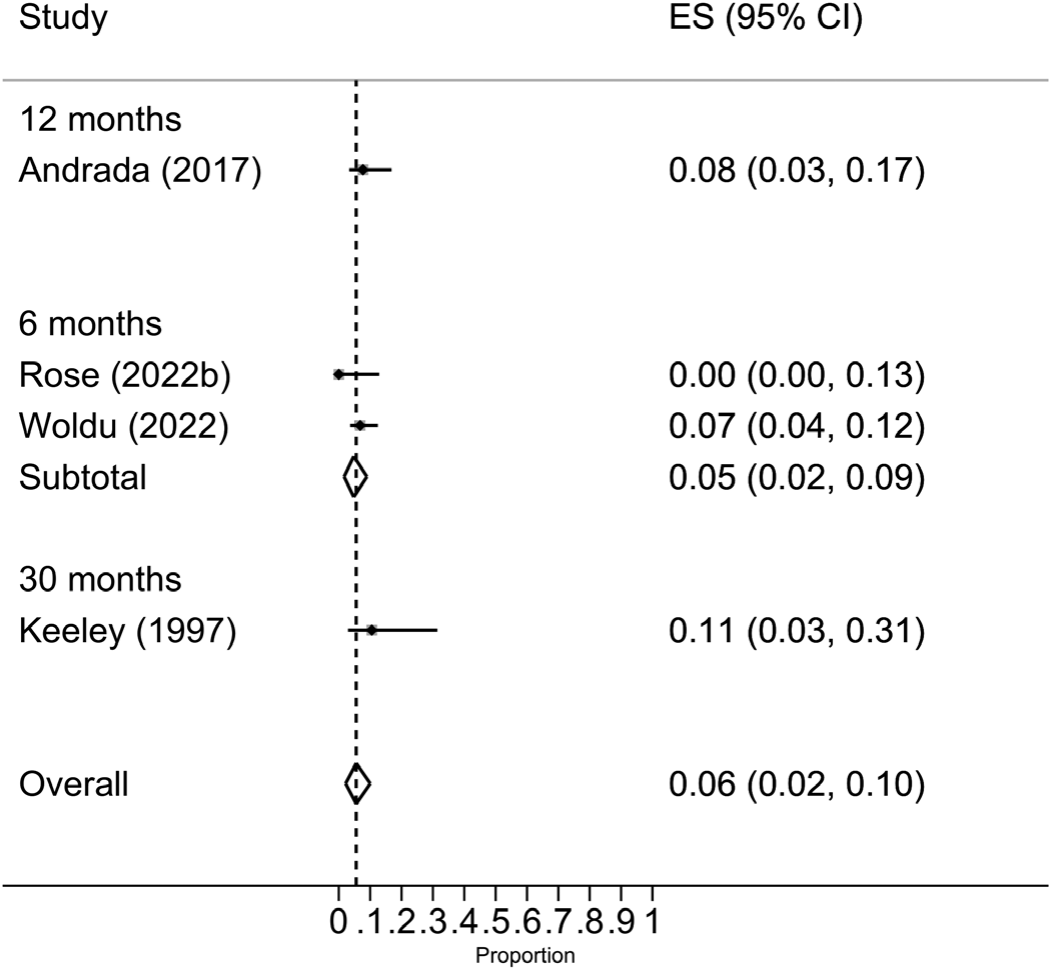
A forest plot showing the pooled prevalence of mortality following UGN-101 therapy in UTUC patients.

Four studies reported the rate of mortality following chemoablation in UTUC (Figure 6). The meta-analysis revealed an overall prevalence rate of mortality of 6% [95% CI: 2%-10%]. The mortality rate differed to a little extent according to the duration of follow-up. For instance, patients had the lowest rate at the earliest timepoint of 6 months (ES = 5%; 95% CI: 2%-9%), and this rate increased progressively at 12 months (ES = 8%; 95% CI: 3%-17%) and 30 months (ES = 11%; 95% CI: 3%-31%) respectively.

### Recurrence

**Figure 7.**
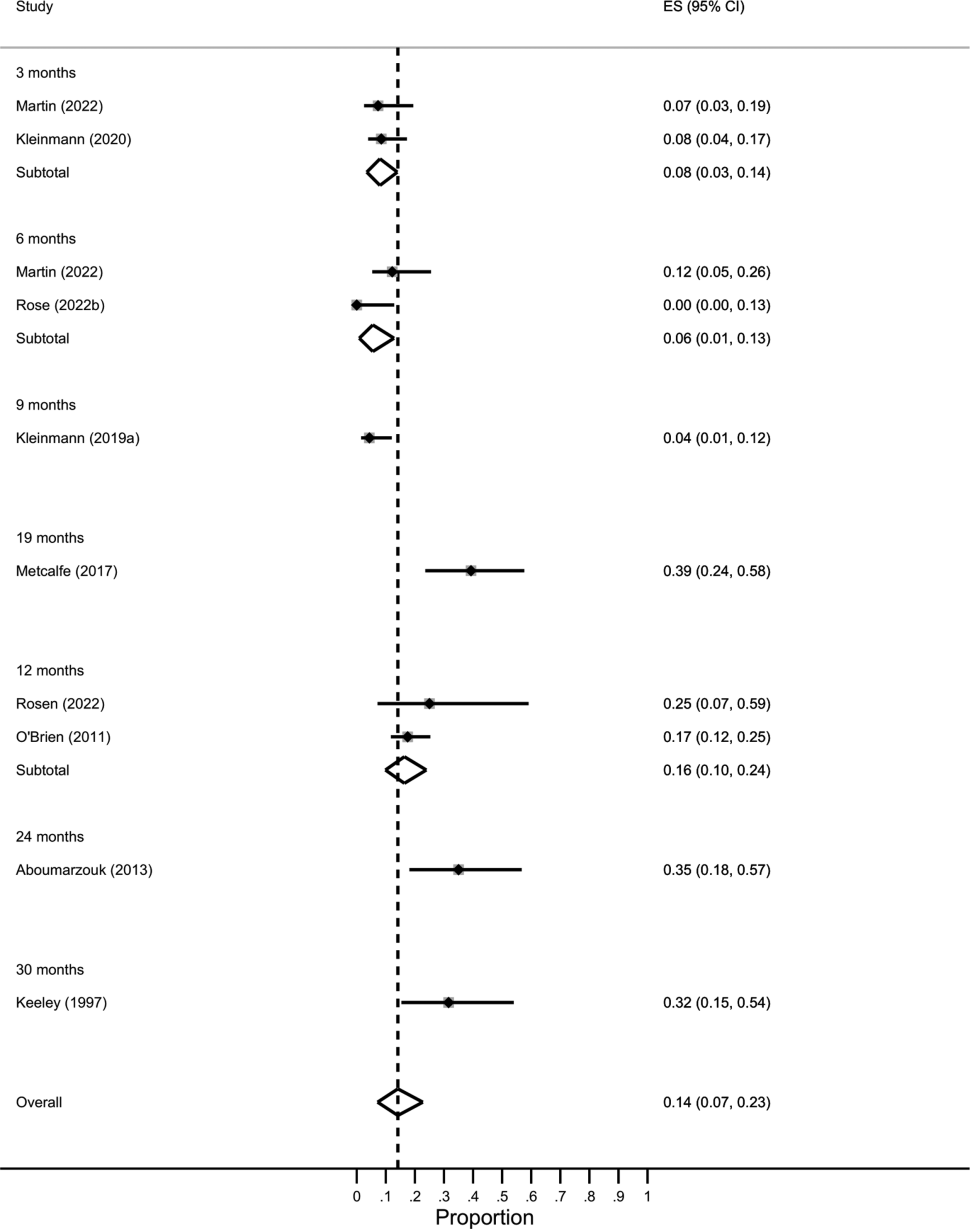
A forest plot showing the pooled prevalence of recurrence following UGN-101 therapy in UTUC patients.

Ten studies reported the recurrence rate of UTUC among patients treated with chemoablation therapy (Figure 7). The overall prevalence of disease recurrence was 14% (95% CI: 7%-23%). This rate was lowest during the early follow-up period and increased drastically on the long term. For example, the rate of recurrence at 3 months of follow-up was 8% (95% CI: 3%-14%) and it reached as high as 39% (95% CI: 24%-58%) during 19 months of follow-up.

### Complications of Chemoablation in UTUC

A summary of all chemoablation-associated complications is provided in Table 4. Overall, 9 studies reported complications following the use of chemoablation among UTUC patients. The meta-analysis revealed an overall rate of 63% ranging from 39% to 85%.

### Based on Follow-up Duration

The rate of complications increased progressively as the duration of the follow-up period increases. At 6 months of follow-up, the rate of complications was 63% (95% CI: 31%-89%), while at 12 months of follow-up the rate was 66% (95% CI: 22%-98%).

### Based on Complication Type

Ten different complications were reported in UTUC patients receiving chemoablation, including ureteral stenosis, urinary tract infection (UTI), hematuria, flank pain, nausea, ileus, delirium, wound infection, fatigue, and sepsis. The meta-analysis showed that ureteral stenosis was the most commonly reported complication, accounting for 30% (95% CI: 2%-58%) of cases, followed by fatigue (ES = 27%; 95% CI: 10%-44%), flank pain (ES = 26%; 95% CI: 16%-36%), UTI (ES = 26%; 95% CI: 12%-39%), and nausea (ES = 24%; 95% CI: 14%-34%) respectively. On the other hand, delirium (ES = 1%; 95% CI: 0%-8%) and wound infection (ES = 1%; 95% CI: 0%-4%) accounted for the least encountered complications.

### Based on Grade and Severity

The majority of reported complications following chemoablation in UTUC patients fell into grades I–II (ES = 59%; 95% CI: 37%-80%) and III (ES = 22%; 95% CI: 10%-36%). Meanwhile, only a minority of patients had grade IV (ES = 1%; 95% CI: 0%-4%) or grade V (ES = 2%; 95% CI: 0%-6%) complications. In the 4 studies reporting serious complications, the meta-analysis revealed a high prevalence of 46% (95% CI: 29%-64%). On the other hand, the occurrence of complications leading to death was rarely encountered, reported in only 1% (95% CI: 0%-7%) of cases.

### Based on the Source of Complication

Three studies reported the source of chemoablation-associated complications. The majority of reported complications were drug-related (ES = 43%; 95% CI: 0%-99%), while only a minority were procedure-related (ES = 14%; 95% CI: 8%-21%).

## Discussion

Therapeutic interventions for UTUC are mainly limited to intracavitary treatments, such as BCG or MMC, often used alongside endoscopic or surgical procedures.^8^ Notably, UGN-101, a novel formulation that combines MMC with RTGel—a temperature-sensitive hydrogel—has gained FDA approval as a primary treatment for patients with low-risk UTUC.^33^ This water-soluble gel, which transitions from liquid at room temperature to gel at body temperature,^16,37^ allows for a tailored application within the patient’s ureteric and pelvicalyceal system.

The topical instillation of MMC is routinely performed following resection of superficial bladder cancer as well as following nephroureterectomy.^10^ Its use has been demonstrated to reduce the recurrence of low-grade disease; however, there is limited evidence for its application in the ureter or renal pelvis. To this end, recent research has investigated the role of intracavitary therapy for low-grade UTUC. This treatment approach of topical instillation has been hypothesized to reduce the incidence of disease recurrence and/or progression.^11^ This outcome may be achieved either through a retrograde approach (i.e., ureteric catheterization), antegrade approach (i.e., percutaneous nephrostomy), or reflux approach (via double-J stent). Although the role of adjuvant intracavitary therapy using chemotherapy has been explored, there is limited evidence that the role of topical MMC in the upper tracts as a primary treatment is low.^12^

A mitomycin C (MMC) gel formulation, UGN-101, has been developed and tested.^13,14^ Given the promising effects of this new formulation the Food and Drug Administration (FDA) issued it a Fast Tract and Orphan Designation status.^15^ As a result, a formal phase III clinical trial, the OLYMPUS trial, was conducted, and the interim results revealed promising clinical outcomes. Four weeks after a 6-week instillation treatment of MMC, 59% of patients showed a complete response regardless of patients’ baseline clinical characteristics.^16^ Based on their findings, it was suggested that MMC could act as a kidney-sparing alternative to UTUC patients with low-grade disease. In their final report, the OLYMPUS trial highlighted that disease recurrence at 12 months occurred in 8 out of 41 UTUC patients; 3 recurrences were documents at 3 months and the remaining 5 occurred at a later stage.^17^

The number of investigations on the role of MMC or UGN-101 gel in UTUC is scarce. This is secondary to the rarity of this type of cancer along with the technical limitations associated with the accessibility of the upper urinary tract for proper instillation of the drug. Most of available data are based on retrospective series; therefore, we conducted this early systematic review to pool all of available evidence on the safety and efficacy of UGN-101 in UTUC patients. We found that UGN-101 was effective in nearly half of the studied population, resulting in a complete response rate of 49%. This rate was highest during the first few months of MMC instillation; however, it started to decline as time passed by. For instance, at 6 months, complete response was at its highest (69%); however, after 12 months of MMC instillation, the rate dropped to 39%. Unfortunately, it was not feasible to examine a time-dependent response to UGN-101 among UTUC patients due to the lack of enough and relevant information. Our results indicate that one fourth of patients had no response; however, this finding should be carefully interpreted given the wide confidence interval of 7 to 49%.

Numerous approaches of MMC instillation into the upper tract of the urinary system have been studied, including the antegrade, retrograde, and reflux approaches. Some of the main barriers associated with the instillation of MMC lie in achieving proper dwell time while ensuring effective drug distribution through the whole urinary collecting system.^38^ The reverse thermal gel containing MMC was designed to provide greater concentration and extended dwell time through which MMC can be in contact with the epithelium of the upper urinary tract in an attempt to reduce MMC absorption through the systemic channels.^39^ In our review, 8 studies reported the antegrade approach using nephrostomy tube, 7 studies reported using the retrograde approach, and only 1 study reported the use of a reflux approach via an indwelling catheter. The remaining studies provided no data as to which method was undertaken. Although a previous report suggested that the use of a double-J stent through the reflux approach provided the least favorable outcomes in terms of efficacy,^40^ the lack of direct head-to-head comparison studies make it difficult to determine which approach is optimal. For this reason, we were unable to conduct a subgroup meta-analysis based on the technique implemented in MMC instillation in UTUC patients. More importantly, we cannot ensure whether the reported outcomes (either positive or negative) were reflective of a true treatment effect or of a poor instillation technique.

It important to highlight that our analysis revealed 13% of patients stopped the treatment. Due to improper reporting, the reasons behind treatment cessation could not be examined. This point needs to be clearly studied in future research. Prior research suggested that MMC instillation was associated with a reduction in the incidence of cancer recurrence and/or progression.^11^ Our study confirms this observation as cancer progression into an advanced stage, in our review, did not surpass the rare event assumption of 5%. This confronts the rate of patients who needed radical nephroureterectomy which accounted for 4% of studies UTUC cases. On the other hand, recurrence was reported in 14% of analyzed cases and this rate increased drastically with time.

The safety of UGN-101 formulation remains one of the most important aspects of chemoablation therapy discussed in the literature. In our study, we noted that 63% of patients had complications, either minor or major. This rate ranged from 39% to 85%, and given this wide interval, more research with larger sample size is still needed to confirm this observation. The duration of follow-up was not a significant effect modifier given the minimal differences in the rates of complications at different time intervals. It should be noted that given the retrospective nature of the majority of included studies, the reported complication rate is liable to assessment and/or reporting bias.

The meta-analysis revealed that ureteral stenosis was the most commonly encountered complication following MMC instillation. This finding denotes that, although MMC is ought to reserve the upper urinary tract, it is associated with high risk of ureteral stenosis (approximately 1 in every 3 patients), reflecting the high toxicity associated with its application. However, this finding is not conclusive and potential to bias given the small patient population analyzed (2 studies, 97 patients) and the lack of accuracy (wide confidence interval ranging from 2% to 58%). This observation should be carefully checked and confirmed in well-designed, larger sample size studies.

Other less frequent complications were fatigue, flank pain, UTI, and nausea in a descending order. The analysis revealed that a 46% of patients had serious complications. This finding should be interpreted with caution. First, analyzed studies did not give any indication as to what ‘serious’ complication referred to. Second, the reported range of serious complications was 29 to 64%, indicating that the true value could lie at any point between both ends of the spectrum (too low or too high rate). Most complications were of grade I to III, while complications grade IV and V occurred only in 1% and 2% of UTUC patients, respectively. The subgroup meta-analysis based on the source of complications highlighted that the procedure itself accounted for a small proportion of reported cases (14%) while MMC administration accounted for 43% of cases. The latter rate should be cautiously interpreted as the confidence interval ranged from 0% to 59%, and this range highlights the imprecision of this finding. This could be related to a number of factors including, but not limited to, the retrospective nature of analyzed studies and the limited number of analyzed studies and the small sample size in each of them.

Based on available evidence, the intracavitary instillation of MMC formulation gel provides an alternative therapeutic option for upper tract urothelial cancer. UGN-101 provides good clinical outcomes in terms of complete response, disease progression and recurrence, and the need to undergo nephroureterectomy. Both serious and non-serious complications were commonly reported; however, they were based on small sample size with lacking accuracy. More well-designed studies with larger sample sizes are still needed to compare the benefits over the risks of MMC use in UTUC.

### Limitations and Future Directions

Although our study provides the biggest evidence on the role of UGN-101 administration as a therapeutic option in UTUC, we came across several limitations during both the qualitative and quantitative synthesis of studied evidence; and thus, our results should not be used for guiding treatment decisions. Rather, our findings are hypothesis-generating and should be used to guide future research in this matter. These limitations include the mixed design (observational and experimental) of included studies along with the small number of patients reported in each study. Although the follow-up duration in some studies reached up to 30 months, the lack of long-term studies highlights the need for future research to examine the long-term efficacy and safety of UGN-101 formulation as a therapeutic option for UTUC. Additionally, future studies should study dose-dependent and approach-dependent comparisons. Finally, given the rarity of UTUC and the subsequent difficulty of conducting randomized clinical trials in this patient cohort, prospective studies are recommended to also investigate the survival outcomes of UTUC patients undergoing MMC instillation.

## Figures and Tables

**Table 1.** The Baseline Characteristics of Studies Reporting the Use of Chemoablation in UTUC Patients

Author (YOP)	Country	Design	Sample Size	Age		Gender		CR Definition	PR Definition
				Mean	SD	Male	Female		
Kleinmann (2020)^26^	USA and Israel	Phase III trial	71	NR	NR	NR	NR	NR	–
Agarwal (2020)^21^	USA	Phase III trial	71	NR	NR	NR	NR	NR	–
Andrada (2017)^41^	Spain	Retrospective cohort	65	67.11	9.4	57	8	–	–
Matin (2022)^17^	USA	Phase III trial (OLYMPUS)	41	70.95	10	27	14	Negative ureteroscopic evaluation, negative cytology, and negative for-cause biopsy	–
Gulamhusein (2020)^23^	UK	Retrospective cohort	69	70	12.11	40	29	–	–
Kleinmann (2019)^25^	USA	Phase III trial (OLYMPUS)	68	NR	NR	NR	NR	Negative ureteroscopic evaluation and negative cytology	–
Kleinmann (2020)^27^	USA	Phase III trial (OLYMPUS)	71	70.4	10	48	23	Negative 3-month ureteroscopic evaluation, negative cytology, and negative for-cause biopsy	–
Kleinmann (2018)^16^	USA	Phase III trial (OLYMPUS)	28	NR	NR	NR	NR	Negative ureteroscopic evaluation and negative wash cytology	–
Kleinmann (2021)^28^	USA	Retrospective cohort	71	NR	NR	NR	NR	–	–
Kleinmann (2019)^15^	USA	Retrospective cohort	16	74.86	9.16	NR	NR	Tumor necrosis or no evidence of neoplasm on cytology or biopsy	Decrease in tumor size with evidence of persistent cancer by cytology or biopsy after completion of treatment
Matin (2021)^30^	USA	Phase III trial (OLYMPUS)	23	70	10.3	0	23	Evaluation was done based on ureteroscopic and local pathology assessment and durability of CR at 12 months	–
Metcalfe (2017)^31^	USA	Retrospective cohort	27	72.21	11.74	16	12	–	–
Rose (2022)^34^	USA	Retrospective cohort	32	74.43	8.69	22	10	–	–
Rose (2022)^33^	USA	Retrospective cohort	26	NR	NR	NR	NR	NR	NR
Rosen (2022)^35^	USA	Retrospective cohort	8	68	6.97	4	4	No radiological or ureteroscopic evidence of disease with negative cytology	Decrease in tumor size by 50% or more.
Woldu (2022)^36^	USA	Retrospective cohort	132	74.46	9.29	93	39	NR	NR
O’Brien (2011)^32^	UK	Phase III tial	120	70.38	9.79	NR	NR	–	–
Aboumarzouk (2013)^20^	Palestine	Retrospective cohort	20	72	7.04	15	4	–	–
Keeley (1997)^24^	USA	Retrospective cohort	19	NR	NR	15	4	No visible tumor, no positive cytology	A reduction in tumor size of more than 50%
Cutress (2012)^22^	UK	Retrospective cohort	73	NR	NR	NR	NR	–	–

“–” indicates that the outcomes were not measured in these studies.

CR, complete response; NR, not reported; PR, partial response; UTUC, upper tract urothelial cancer; YOP, Year of Publication (although it was a measured outcome).

**Table 2.** The Characteristics of UTUC and MMC Instillation Among Patients Receiving Chemoablation

Author (YOP)	Tumor Characteristics			MMC Instillation	
	**History of UTUC**	**UTUC Location**	UTUC Size*	**Approach**	**Maintenance**
Kleinmann (2020)^26^	NR	NR	NR	Retrograde catheter [N = 71]	No
Agarwal (2020)^21^	NR	NR	NR	Retrograde instillation [N = 71]	Yes
Andrada (2017)^41^	NR	Renal calyces [N = 3]; renal pelvis [N = 22]; ureter [N = 29]; others [N = 22]	11.12 (5.34)	Nephrostomy tube [N = 18]; Ureteral catheter [N = 2]	No
Matin (2022)^17^	20	NR	13.93 (9.58)	Retrograde instillation [N = 41]	Yes
Gulamhusein (2020)^23^	NR	NR	NR	Through the indwelling catheter [N = 69]	No
Kleinmann (2019)^25^	NR	NR	NR	Retrograde catheter [N = 68]	No
Kleinmann (2020)^27^	37	NR	14.8 (8.7)	Ureteral catheter [N = 71]	No
Kleinmann (2018)^16^	NR	NR	NR	Retrograde catheter [N = 28]	No
Kleinmann (2021)^28^	NR	NR	NR	NR	No
Kleinmann (2019)^15^	NR	NR	NR	Percutaneous nephrostomy tube [N = 16]	Yes
Matin (2021)^30^	10	NR	NR	NR	Yes
Metcalfe (2017)^31^	NR	NR	NR	Nephrostomy [N = 9]; Ureteral catheter [N = 19]	No
Rose (2022)^34^	NR	Upper pole [N = 8]; interpolar [N = 1]; lower pole [N = 6]; renal pelvis [N = 2]; ureter [N = 1]; multifocal [N = 14]	2 (1.55)	Antegrade endoscopic approach [N = 4]; Retrograde endoscopic approach [N = 28]	No
Rose (2022)^33^	NR	NR	NR	Percutaneous nephrostomy tube [N = 26]	Yes
Rosen (2022)^35^	NR	NR	NR	Antegrade endoscopic approach [N = 8]	No
Woldu (2022)^36^	NR	NR	NR	Antegrade instillation via a percutaneous nephrostomy (PCN) tube [N = 56]; Retrograde instillation in the clinic [N = 48]; Retrograde instillation under anesthesia [N = 28]	No
O’Brien (2011)^32^	NR	NR	NR	NR	
Aboumarzouk (2013)^20^	NR	Renal pelvis [N = 9]; lower ureter [N = 10]; mid ureter [N = 1]	NR	5F open-ended ureteric catheter [N = 20]	No
Keeley (1997)^24^	NR	NR	NR	6F open-ended ureteral catheter [N = 19]	No
Cutress (2012)^22^	NR	NR	NR	Nephrostomy tube [N = 73]	No

NR, not reported; UTUC, upper tract urothelial cancer; YOP, year of publication; MMC, mitomycin C.

*Data are presented in the form of mean (SD).

**Table 3.** The Risk of Bias of Each of each of the included studies using the Newcastle Ottawa Scale

Author (YOP)	Selection	Comparability	Exposure	Overall Quality
Kleinmann (2020)^26^	3	2	2	Fair
Agarwal (2020)^21^	3	2	2	Fair
Andrada (2017)^41^	3	2	2	Fair
Matin (2022)^17^	3	2	2	Fair
Gulamhusein (2020)^23^	3	2	2	Fair
Kleinmann (2019)^25^	3	2	2	Fair
Kleinmann (2020)^27^	3	2	2	Fair
Kleinmann (2018)^16^	3	2	2	Fair
Kleinmann (2021)^28^	3	2	2	Fair
Kleinmann (2019)^15^	3	2	2	Fair
Matin (2021)^30^	3	2	2	Fair
Metcalfe (2017)^31^	2	2	2	Poor
Rose (2022)^34^	3	2	2	Fair
Rose (2022)^33^	3	2	2	Fair
Rosen (2022)^35^	3	2	2	Fair
Woldu (2022)^36^	3	2	2	Fair
O’Brien (2011)^32^	3	2	2	Fair
Aboumarzouk (2013)^20^	3	2	2	Fair
Keeley (1997)^24^	3	2	2	Fair
Cutress (2012)^22^	2	2	2	Poor

YOP, year of publication.

**Table 4.** A Summary of Reported Complications Based on the Grade, Follow-up Time, and Source Among UTUC Patients Receiving Chemoablation

Outcome	Subgroup	Studies (N)	Patients (N)	Effect Estimate	95% CI
Complications based on follow-up					
	6 months	3	166	63%	31%-89%
	12 months	4	204	66%	22%-98%
	15 months	1	32	94%	80%-98%
	24 months	1	20	15%	5%-36%
	Overall	9	422	63%	39%-85%
Complications’ type					
	Ureteral stenosis	3	168	30%	2%-58%
	UTI	3	168	26%	12%-39%
	Hematuria	2	97	19%	4%-42%
	Flank pain	2	97	26%	16%-36%
	Nausea	1	71	24%	14%-34%
	Ileus	1	69	7%	1%-13%
	Delirium	1	69	1%	0%-8%
	Wound infection	1	69	1%	0%-4%
	Fatigue	1	26	27%	10%-44%
	Sepsis	1	26	8%	0%-18%
Complications’ grade/stage					
	Grades I–II	3	111	59%	37%-80%
	Grade III	4	131	22%	10%-36%
	Grade IV	2	103	1%	0%-4%
	Grade V	2	103	2%	0%-6%
	Serious	4	151	46%	29%-64%
	Leading to death	3	144	1%	0%-7%
Source of complications					
	Drug-related	3	126	43%	0%-99%
	Procedure-related	2	110	14%	8%-21%

CI, confidence interval; N, number; UTI, urinary tract infection; UTUC, upper tract urothelial cancer.

**Supplementary Table 1.** The Detailed Search Criteria Used in Each of the Searched Databases

Database	No	Search Query	Results
**PubMed**			
	#1	Chemoablation OR UGN-101 OR “mitomycin gel” OR “mitomycin-containing”	99
	#2	“upper tract” OR “upper urinary tract”	10713
	#3	“urothelial cancer” OR “urothelial carcinoma*” OR “urothelial neoplasm” OR “transitional cell carcinoma”	31778
	#4	#2 AND #3	3923
	#5	Nephroureterectomy OR “Nephroureterectomy”[Mesh] OR “Ureteral Neoplasms”[Mesh]	7472
	#6	#4 OR #5	8982
	#7	#6 AND #1	14
**Scopus**			
	#1	ALL(Chemoablation) OR ALL(UGN-101) OR ALL(“mitomycin gel”) OR ALL(“mitomycin-containing”)	506
	#2	ALL(“upper tract”) OR ALL(“upper urinary tract”)	36275
	#3	ALL(“urothelial cancer”) OR ALL(“urothelial carcinoma”) OR ALL(“urothelial neoplasm”) OR ALL(“transitional cell carcinoma”)	108139
	#4	#2 AND #3	13784
	#5	ALL(Nephroureterectomy)	9081
	#6	#4 OR #5	18024
	#7	#6 AND #1	84
**Web of Science**			
	#1	ALL=Chemoablation OR ALL=UGN-101 OR ALL=”mitomycin gel” OR ALL=”mitomycin-containing”	115
	#2	ALL=”upper tract” OR ALL=”upper urinary tract”	10926
	#3	ALL=”urothelial cancer” OR ALL=”urothelial carcinoma*” OR ALL=”urothelial neoplasm” OR ALL=”transitional cell carcinoma”	33711
	#4	#2 AND #3	5144
	#5	ALL=Nephroureterectomy	3979
	#6	#4 OR #5	6735
	#7	#6 AND #1	20
**CENTRAL**			
	#1	Chemoablation OR UGN-101 OR “mitomycin gel” OR “mitomycin-containing”	33
	#2	“upper tract” OR “upper urinary tract”	589
	#3	“urothelial cancer” OR “urothelial carcinoma*” OR “urothelial neoplasm” OR “transitional cell carcinoma”	1682
	#4	#2 AND #3	199
	#5	Nephroureterectomy	131
	#6	#4 OR #5	225
	#7	#6 AND #1	3
**EBSCOhost - Academic Search Complete**			
	#1	TX Chemoablation OR TX UGN-101 OR TX mitomycin	18362
	#2	TX “upper tract” OR TX “upper urinary tract”	8747
	#3	TX “urothelial cancer” OR TX “urothelial carcinoma” OR TX “urothelial neoplasm” OR TX “transitional cell carcinoma”	20525
	#4	#2 AND #3	3299
	#5	TX Nephroureterectomy	2112
	#6	#4 OR #5	3299
	#7	#6 AND #1	394
**Clinicaltrials.gov**			
	#1	Urothelial carcinoma	76
	#2	UGN-101 OR chemoablation OR mitomycin	37
	#3	#1 AND #2 [complete + with results]	5
**Google Scholar**			
	With all of the words	urothelial upper tract	
	With the exact phrase		
	With at least one of the words	UGN-101 chemoablation	
	Total	Only the first 200 were retrieved and screened	200

No: Number; TX: full text

## References

[1] Oldbring J Glifberg I Mikulowski P Hellsten S . Carcinoma of the renal pelvis and ureter following bladder carcinoma: frequency, risk factors and clinicopathological findings. J Urol. 1989;141(6):1311 1313. (10.1016/s0022-5347(17)41291-2) 2724427

[2] Rabbani F Perrotti M Russo P Herr HW . Upper-tract tumors after an initial diagnosis of bladder cancer: argument for long-term surveillance. J Clin Oncol. 2001;19(1):94 100. (10.1200/JCO.2001.19.1.94) 11134200

[3] Picozzi S Ricci C Gaeta M , et al. Upper urinary tract recurrence following radical cystectomy for bladder cancer: a meta-analysis on 13,185 patients. J Urol. 2012;188(6):2046 2054. (10.1016/j.juro.2012.08.017) 23083867

[4] Tran W Serio AM Raj GV , et al. Longitudinal risk of upper tract recurrence following radical cystectomy for urothelial cancer and the potential implications for long-term surveillance. J Urol. 2008;179(1):96 100. (10.1016/j.juro.2007.08.131) 17997449

[5] Gadzinski AJ Roberts WW Faerber GJ Wolf JS Jr . Long-term outcomes of nephroureterectomy versus endoscopic management for upper tract urothelial carcinoma. J Urol. 2010;183(6):2148 2153. (10.1016/j.juro.2010.02.005) 20399468

[6] Margulis V Shariat SF Matin SF , et al. Outcomes of radical nephroureterectomy: a series from the Upper Tract Urothelial Carcinoma Collaboration. Cancer. 2009;115(6):1224 1233. (10.1002/cncr.24135) 19156917

[7] Urology EAo. EAU Guidelines on Upper Urinary Tract Urothelial Cell Carcinoma. European Association of Urology; 2022.

[8] Khargi R Connors C Ricapito A , et al. Adjuvant intraluminal therapies for upper tract urothelial carcinoma. Transl Androl Urol. 2023;12(9):1439 1448. (10.21037/tau-23-35) 37814698 PMC10560344

[9] Numakura K Miyake M Kobayashi M , et al. Subsequent upper urinary tract carcinoma related to worse survival in patients treated with BCG. Cancers. 2023;15(7):2002. (10.3390/cancers15072002) 37046663 PMC10092972

[10] Babjuk M Burger M Capoun O , et al. European Association of Urology guidelines on non–muscle-invasive bladder cancer (Ta, T1, and carcinoma in situ). Eur Urol. 2022;81(1):75 94. (10.1016/j.eururo.2021.08.010) 34511303

[11] Leow JJ Liu Z Tan TW Lee YM Yeo EK Chong YL . Optimal management of upper tract urothelial carcinoma: current perspectives. Onco Targets Ther. 2020;13:1 15. (10.2147/OTT.S225301) 32021250 PMC6954076

[12] Cutress ML Stewart GD Zakikhani P Phipps S Thomas BG Tolley DA . Ureteroscopic and percutaneous management of upper tract urothelial carcinoma (UTUC): systematic review. BJU Int. 2012;110(5):614 628. (10.1111/j.1464-410X.2012.11068.x) 22471401

[13] Donin N Duarte S Strauss-Ayali D , et al. PD13-10 preclinical trial of serial MITOGEL® instillations into the pelvicalyceal system of the Yorkshire swine. J Urol. 2016;195(4S):e301-e. (10.1016/j.juro.2016.02.981)

[14] Meiron M Chamie K Lerner SP , et al. MP77-08 MITOGEL: optimizing drug delivery to the upper urinary TRACT—A preclinical evaluation. J Urol. 2014;191(4S):e914-e. (10.1016/j.juro.2014.02.2471)

[15] Kleinmann N Wirth G Lin JS , et al. Thermo reversible hydrogel based delivery of Mitomycin C (UGN-101) for treatment of upper tract urothelial carcinoma (UTUC). Bladder Cancer. 2019;5(1):21 29. (10.3233/BLC-180182)

[16] Kleinmann N Pierorazio P Matin S , et al. LBA25 non-surgical management of low grade upper tract urothelial cancer: an interim analysis of the international multicenter Olympus trial (NCT02793128). J Urol. 2018;199(4S):e1166. (10.1016/j.juro.2018.03.097)

[17] Matin SF Pierorazio PM Kleinmann N , et al. Durability of response to primary chemoablation of low-grade upper tract urothelial carcinoma using UGN-101, a mitomycin-containing reverse thermal gel: Olympus trial final report. J Urol. 2022;207(4):779 788. (10.1097/JU.0000000000002350) 34915741 PMC12721675

[18] Amir-Behghadami M Janati A . Population, Intervention, Comparison, Outcomes and Study (PICOS) design as a framework to formulate eligibility criteria in systematic reviews. Emerg Med J. 2020;37(6):387. (10.1136/emermed-2020-209567) 32253195

[19] Muka T Glisic M Milic J , et al. A 24-step guide on how to design, conduct, and successfully publish a systematic review and meta-analysis in medical research. Eur J Epidemiol. 2020;35(1):49 60. (10.1007/s10654-019-00576-5) 31720912

[20] Aboumarzouk OM Somani B Ahmad S Nabi G Townell N Kata SG . Mitomycin C instillation following ureterorenoscopic laser ablation of upper urinary tract carcinoma. Urol Ann. 2013;5(3):184 189. (10.4103/0974-7796.115746) 24049383 PMC3764901

[21] Agarwal PK Sternberg CN . Clinical trials corner Issue 8 (1). Bladder Cancer. 2022 [preprint]:1 3. 38994518 10.3233/BLC-229001 PMC11181851

[22] Cutress ML Stewart GD Wells‐Cole S Phipps S Thomas BG Tolley DA . Long‐term endoscopic management of upper tract urothelial carcinoma: 20‐year single‐centre experience. BJU Int. 2012;110(11):1608 1617. (10.1111/j.1464-410X.2012.11169.x) 22564677

[23] Gulamhusein A Silva P Cullen D , et al. Safety and feasibility of early single‐dose Mitomycin C bladder instillation after robot‐assisted radical nephroureterectomy. BJU Int. 2020;126(6):739 744. (10.1111/bju.15162) 32638490

[24] Keeley FX Jr Bagley DH . Adjuvant Mitomycin C following endoscopic treatment of upper tract transitional cell carcinoma. J Urol. 1997;158(6):2074 2077. (10.1016/s0022-5347(01)68157-6) 9366315

[25] Kleinmann N Matin S Pierorazio P , et al. LBA-16 nephron-sparing management of low grade (LG) UTUC with UGN-101 (mitomycin gel) for instillation: the Olympus trial experience. J Urol. 2019;201(suppl 4):e999-e. (10.1097/01.JU.0000557508.23335.0a)

[26] Kleinmann N Matin S Pierorazio P , et al. PD18-07 primary chemoablation for the treatment of low grade upper tract urothelial carcinoma: the Olympus trial. J Urol. 2020;203(suppl 4):e378-e. (10.1097/JU.0000000000000861.07) PMC12721675 34915741

[27] Kleinmann N Matin SF Pierorazio PM , et al. Primary chemoablation of low-grade upper tract urothelial carcinoma using UGN-101, a mitomycin-containing reverse thermal gel (Olympus): an open-label, single-arm, phase 3 trial. Lancet Oncol. 2020;21(6):776 785. (10.1016/S1470-2045(20)30147-9) 32631491

[28] Kleinmann N Pierorazio P Raman J , et al. LBA02-10 long-term recurrence free survival following UGN-101 treatment for low-grade upper tract urothelial carcinoma. J Urol. 2021;206(suppl 3):e1179. (10.1097/JU.0000000000002149.10)

[29] Labbate C Woldu S Murray K , et al. Efficacy and safety of mitomycin gel (UGN-101) as an adjuvant therapy after complete endoscopic management of upper tract urothelial carcinoma. J Urol. 2023;209(5):872 881. (10.1097/JU.0000000000003185) 36657029

[30] Matin S Smith A Linehan J , et al. MP48-18 female patients with low-grade upper tract urothelial carcinoma: primary chemoablation and durability of response in a subgroup analysis from the Olympus trial. J Urol. 2021;206(suppl 3):e872. (10.1097/JU.0000000000002074.18)

[31] Metcalfe M Wagenheim G Xiao L , et al. Induction and maintenance adjuvant Mitomycin C topical therapy for upper tract urothelial carcinoma: tolerability and intermediate term outcomes. J Endourol. 2017;31(9):946 953. (10.1089/end.2016.0871) 28731777 PMC5915259

[32] O’Brien T Ray E Singh R Coker B Beard R , British Association of Urological Surgeons Section of Oncology. Prevention of bladder tumours after nephroureterectomy for primary upper urinary tract urothelial carcinoma: a prospective, multicentre, randomised clinical trial of a single postoperative intravesical dose of Mitomycin C (the ODMIT-C Trial). Eur Urol. 2011;60(4):703 710. (10.1016/j.eururo.2011.05.064) 21684068

[33] Rose K Narang G Rosen G , et al. PD58-06 antegrade administration of reverse thermal mitomycin gel for primary chemoablation of upper tract urothelial carcinoma via percutaneous nephrostomy tube: a multi-institutional real-world experience. J Urol. 2022;207(suppl 5):e1013. (10.1097/JU.0000000000002643.06)

[34] Rose KM Narang G Rosen G , et al. Antegrade administration of mitomycin gel for upper tract urothelial carcinoma via percutaneous nephrostomy tube: a multi‐institutional retrospective cohort study. BJU Int. 2023;131(4):471 476. (10.1111/bju.15925) 36285629

[35] Rosen GH Nallani A Muzzey C Murray KS . Antegrade instillation of UGN-101 (mitomycin for pyelocalyceal solution) for low-grade upper tract urothelial carcinoma: initial clinical experience. J Urol. 2022;207(6):1302 1311. (10.1097/JU.0000000000002454) 35130080

[36] Woldu SL Labbate C Murray KS , et al., eds. Early experience with UGN-101 for the treatment of upper tract urothelial cancer–A multicenter evaluation of practice patterns and outcomes. Urol Oncol. Elsevier; Amsterdam. 2023;41(3):147.e15 147.e21. (10.1016/j.urolonc.2022.10.029) 36424224

[37] Lin JS-J Kleinmann N Wirth GJ , et al. Thermo Reversible Hydrogel Based Delivery of Mitomycin C for Treatment of Upper Tract Urothelial Carcinoma (UTUC). American Society of Clinical Oncology; 2017.

[38] Donin NM Duarte S Lenis AT , et al. Sustained-release formulation of Mitomycin C to the upper urinary tract using a thermosensitive polymer: a preclinical study. Urology. 2017;99:270 277. (10.1016/j.urology.2016.09.039) 27720772

[39] Shabsigh A Kleinmann N Smith AB , et al. Pharmacokinetics of UGN-101, a mitomycin-containing reverse thermal gel instilled via retrograde catheter for the treatment of low-grade upper tract urothelial carcinoma. Cancer Chemother Pharmacol. 2021;87(6):799 805. (10.1007/s00280-021-04246-w) 33677615 PMC8110485

[40] Yossepowitch O Lifshitz DA Dekel Y , et al. Assessment of vesicoureteral reflux in patients with self-retaining ureteral stents: implications for upper urinary tract instillation. J Urol. 2005;173(3):890 893. (10.1097/01.ju.0000147747.89028.64) 15711312

[41] Andrada AO García IL Fúnez FA , et al. Conservative treatment of upper urinary tract carcinoma: long-term results. Can Urol Assoc J. 2017;11(7):E291 E296. (10.5489/cuaj.4173) PMC5519388 28761590

